# PTSD and complex PTSD in sentenced male prisoners in the UK: prevalence, trauma antecedents, and psychiatric comorbidities

**DOI:** 10.1017/S0033291720004936

**Published:** 2022-10

**Authors:** Emma Facer-Irwin, Thanos Karatzias, Annie Bird, Nigel Blackwood, Deirdre MacManus

**Affiliations:** 1 Researcher; Forensic and Neurodevelopmental Sciences Department, Institute of Psychiatry, Psychology and Neuroscience, King's College London, London, England; 2 Professor of Mental Health; School of Health & Social Care, Edinburgh Napier University, Edinburgh, Scotland; 3 Clinical & Health Psychologist; Rivers Centre for Traumatic Stress, NHS Lothian, Edinburgh, Scotland; 4 Research Assistant; Forensic and Neurodevelopmental Sciences Department, Institute of Psychiatry, Psychology and Neuroscience, King's College London, London, England; 5 Clinical Reader in Forensic Psychiatry; Forensic and Neurodevelopmental Sciences Department, Institute of Psychiatry, Psychology and Neuroscience, King's College London, London, England; 6 Consultant Forensic Psychiatrist; HMP Wandsworth, South London & Maudsley NHS Foundation Trust, London, England; 7 Consultant Forensic Psychiatrist; London and South East NHS Veterans’ Mental Health Service, Camden and Islington NHS Trust; HMP Wandsworth, South London and Maudsley NHS Trust, London, England

**Keywords:** Complex posttraumatic stress disorder, ICD-11, prison, trauma-informed care

## Abstract

**Background:**

Posttraumatic stress disorder (PTSD) is highly prevalent within prison settings, yet is often unidentified and undertreated. Complex PTSD (CPTSD) has been recently formally recognised in the International Classification of Diseases 11th revision (ICD-11) diagnostic framework but has never been explored in prison settings. We aimed to establish the prevalence of ICD-11 PTSD and CPTSD in a UK prison sample using a validated instrument (the International Trauma Questionnaire). We also explored the associations of these two diagnoses with their traumatic antecedents and psychiatric comorbidities.

**Method:**

Randomly selected male, sentenced prisoners in a large medium-security prison in south London (*N* = 221) took part in a clinical interview which assessed PTSD, CPTSD, trauma histories, and comorbid disorders. Multinomial logistic regression was performed to examine differences between those with PTSD or CPTSD, and those without symptoms.

**Results:**

A total of 7.7% (95% CI 4.5–12) of the male sentenced prisoners met diagnostic criteria for ICD-11 PTSD and 16.7% (95% CI 12.1–22.3) for CPTSD. A diagnosis of PTSD was associated with more recent traumatic exposure, comorbid generalised anxiety disorder, alcohol dependence, and Cluster B personality disorder. A diagnosis of CPTSD was associated with complex trauma exposure antecedents (developmental, interpersonal, repeated, or multiple forms), and comorbid with anxiety, depression, substance misuse, psychosis, and ADHD.

**Conclusions:**

This study confirms that CPTSD is a very common and comorbid condition in male prisoners. There is an urgent need to develop trauma-informed care in prisons.

## Introduction

Posttraumatic stress disorder (PTSD) is more prevalent in prison than in community samples (Baranyi, Cassidy, Fazel, Priebe, & Mundt, 2018). Studies in prison populations suggest substantial psychiatric comorbidity, associations with suicidal and aggressive behaviour (Facer-Irwin et al., 2019) and considerable unmet treatment needs (Jakobowitz et al., 2017; Tyler, Miles, Karadag, & Rogers, 2019). A small number of studies in the UK have sought to establish the prevalence of PTSD in prisoners, with estimates ranging widely from 1.7 to 13.9% (Bebbington et al., 2017; Brooke, Taylor, Gunn, & Maden, 1996; Tyler et al., 2019). However, these previous studies have not considered trauma aetiology, nor have they distinguished between PTSD and complex PTSD (CPTSD). CPTSD has been recently introduced into the ICD-11 diagnostic framework and is a disorder characterised by PTSD symptomatology as well as additional difficulties in emotional regulation, negative alterations in the perception of self, and relationship disturbances (Brewin et al., 2017). Substantial evidence suggests that men and women in prison experience high rates of the types of complex developmental traumas which have been shown to increase the risk of developing CPTSD (Howard, Karatzias, Power, & Mahoney, 2017; Karatzias et al., 2018; Wolff, Huening, Shi, & Frueh, 2014). However, no study to date has explored the prevalence and traumatic antecedents of these two sibling disorders using validated instruments in a prison sample or examined the psychiatric comorbidity of both conditions (Facer-Irwin et al., 2019). Thus, we sought to explore the prevalence of PTSD and CPTSD in a male sentenced prisoner population using the first validated diagnostic tool for the newly formulated ICD-11 diagnoses of PTSD and CPTSD, namely the International Trauma Questionnaire (Cloitre et al., 2018). We additionally explored the associations of these two diagnoses with a range of traumatic antecedents and distinct psychiatric conditions.

## Method

### Sample

The study population consisted of sentenced male prisoners between the ages of 18 and 55 who had recently arrived into custody (in the week prior to sampling) at a large (prisoner capacity = 1650) Category B (medium-security) prison in south London, UK. The cross-sectional data explored in this paper were collected as part of a wider prospective cohort study examining the impact of PTSD on behavioural outcomes amongst the male prison population. Due to the logistic complications of collecting data simultaneously across multiple sites, a sample of female prisoners could not be included.

Sampling occurred weekly, commencing the week of 17 July 2017 and ending 1 March 2019. During this period, 9075 prisoners were received into custody, of whom 3477 met initial eligibility criteria. Potentially eligible participants were identified from prison reception lists. The sample was stratified according to prisoner status, and included participants who were: newly sentenced, arriving from court; serving sentences, transferred from other prisons; on license and recalled back to prison from the community. Potential participants were then randomly selected using a random number generating process and approached on prison wings by a researcher to obtain informed consent. We approached 12% (*n* = 432) of eligible prisoners during the sampling timeframe. Our initial participation rate (defined as the proportion of those approached who were eligible and consented to take part) was 75%. The final sample *N* in this study was 221 male sentenced prisoners. For a detailed description of study recruitment procedures and exclusion criteria, please see the recruitment flowchart in the supplementary material.

Ethical approval was gained for access to non-identifiable demographic data (ethnicity, age, and main offence) from prison reception lists. This allowed for differences to be identified between individuals who participated and those who declined to participate in the study (*n* = 88, 20%), and to weight analyses for any potential non-response biases. Preliminary analysis indicated that participants did not differ from non-participants in terms of ethnicity (*X*^2^ = 0.03, *p* = 0.86), age (*t* = −0.083, *p* = 0.41), or commission of a violent index offence (*X*^2^ = 0.05, *p* = 0.82).

### Procedure

All procedures contributing to this work complied with the ethical standards of the relevant national and institutional committees on human experimentation and with the Helsinki Declaration of 1975, as revised in 2008. All procedures were approved by NHS England (Ref: 16/SS/0179) and the National Offender Management Service (Ref: 2016-321).

Recruitment and interview procedures were conducted within approximately 2 weeks of the prisoner's arrival into custody. Written informed consent was obtained from all subjects. Consenting participants took part in a clinical interview with a researcher. As literacy and educational attainment levels in prison are often low (Singleton, Meltzer, Gatward, Coid, & Deasy, 1998), all self-report questionnaires included in this assessment were administered in an interview format. Interviews were conducted in private rooms on the prison wings. Interviewers were postgraduate-level researchers in psychology (MSc level or above), with several years of experience working in forensic settings. All interviewers received specific training in the administration of each of the tools involving several weeks of observation, followed by practice and in-vivo training sessions led by a senior consultant psychiatrist (DM) and/or the lead researcher (EFI) prior to data collection. Initial assessments were observed by the lead researcher and further spot checks conducted throughout the project for quality assurance and standardisation purposes. All measures were administered once only.

### Measures

#### PTSD and CPTSD (the International Classification of Diseases 11th revision)

The International Trauma Questionnaire (ITQ) (Cloitre et al., 2018) is the only validated tool to assess for current (past month) PTSD and CPTSD according to the International Classification of Diseases 11th revision (ICD-11) diagnostic criteria (World Health Organization, 2018), and has demonstrated good psychometric properties (Cloitre et al., 2018; Karatzias et al., 2017). The ITQ consists of 18 items. Twelve of these items assess current (past month) symptoms – six items assess the three PTSD symptom clusters (re-experiencing, avoidance, and sense of threat), and six items assess the three CPTSD symptom clusters described as disturbances in self-organisation (DSO) – affect dysregulation, negative self-concept, and disturbed relationships. Six additional items assess functional impairment in three dimensions (work, relationships, or other meaningful activities).

All items assess the presence of symptomology using a Likert scale (0–4) from ‘not at all’ to ‘extremely.’ A traumatic event meeting ICD-11 criteria, defined as ‘a threatening or horrific event or series of events’, was required for both diagnoses (WHO, 2018). A ‘probable’ diagnosis of PTSD or CPTSD was then determined if an individual met the symptom threshold (⩾2) on at least one item within each symptom cluster, plus the endorsement of functional impairment in at least one domain. Those with CPTSD first met PTSD criteria, as well as criteria for additional DSO symptoms. In the ICD-11, an individual may have either PTSD or CPTSD, but not both (Cloitre et al., 2018). Cronbach's alpha of the ITQ in the current sample was 0.92 for PTSD items, and 0.90 for CPTSD items, indicating strong reliability.

#### Trauma

The trauma characteristics explored in this study were: trauma type, the timing of trauma exposure (recency of index event; occurring in childhood or adulthood), duration of index trauma exposure (single-incident *v.* repeated exposure), and cumulative trauma exposure (exposure to multiple forms of trauma over time). Information regarding trauma exposures came from three retrospective self-report sources: the Adverse Childhood Experiences (ACE) questionnaire (Felitti et al., 1998) to measure childhood adverse life events; the Life Events Checklist (LEC) (Weathers et al., 2013) to measure trauma across the lifespan; and the ITQ to measure the antecedent or ‘worst’ trauma, as identified by participants during the assessment of PTSD/CPTSD, with which the associated symptoms correspond. Data on antecedent trauma was therefore missing for the 20 participants who either did not report an index traumatic event (i.e. not trauma-exposed) or reported one which did not meet ICD-11 criteria. Cumulative childhood trauma and/or adversity was determined using an established research cut-off (⩾5 ACEs), while cumulative lifetime trauma was defined categorically using a recommended threshold specific to the sample being investigated – namely one-above the mean of trauma types experienced by the entire sample (Ford & Delker, 2018). A table outlining the definition and measure(s) used for each trauma characteristic can be found in the supplementary material.

#### Comorbid mental disorders

Other current mental disorders measured by this study were depression, generalised anxiety, substance abuse, alcohol abuse and dependence, psychosis, mania, antisocial personality disorder, borderline personality disorder, and ADHD. Symptoms of depression and generalised anxiety were measured using the Patient Health Questionnaire-9 item (PHQ-9) (Kroenke, Spitzer, & Williams, 2001) and the Generalised Anxiety Disorder-7 item questionnaire (GAD-7) (Spitzer, Kroenke, Williams, & Lowe, 2006), well-validated tools used previously amongst prison populations (Evans et al., 2017). For both disorders, the more restrictive cut-off scores of ⩾15 were used to establish probable diagnoses. Probable substance abuse was measured using the Drug Abuse Screening Test (DAST) with a recommended cut off score of ⩾6 (Skinner, 1982). Harmful alcohol use and alcohol dependence were measured using the Alcohol Use Disorders Identification Test (AUDIT) with cut off scores of 16–19 and 20 or higher, respectively (Babor, de la Fuente, Saunders, & Grant, 2001). Both measures have been previously used to measure substance and alcohol problems among prisoners (Capuzzi et al., 2020; MacAskill et al., 2011). Probable ADHD was established using the Adult ADHD Self-report Scale (ASRS), a well-validated six-item self-report scale developed by the WHO and previously used in prison research (Ginsberg, Hirvikoski, & Lindefors, 2010), with probable ADHD estimated using threshold cut-off scores for each symptom question (Kessler et al., 2005). Mania, psychosis, and antisocial personality disorder were all assessed by the Mini International Neuropsychiatric Interview (MINI) (Lecrubier et al., 1997), a structured diagnostic interview used frequently in previous prison studies (Fazel, Hayes, Bartellas, Clerici, & Trestman, 2016). In accordance with recommendations from a previous prison study (Marzano, Fazel, Rivlin, & Hawton, 2010), due to concerns that the MINI may over-diagnose mania in custodial settings (Fazel et al., 2016), one adjustment was made to this schedule: a diagnosis of mania was only made when participants met criteria for elation/expansiveness (i.e. irritable mood alone was insufficient to reach a diagnosis). As the MINI does not include a measure of Borderline Personality Disorder, this was assessed using the SCID-5 (First, Williams, Karg, & Spitzer, 2015) BPD module, introduced mid-way through data collection and measured on a sub-sample of the total population (*n* = 101). ASPD and BPD were found to be highly comorbid within our sample – only three individuals with BPD did not also have comorbid ASPD. As assessing BPD individually in regression models would have restricted analysis in that group, and collinearity prevented these diagnoses from being assessed in the same model, a Cluster B Personality Disorder category was created, whereby 1 = either ASPD and/or BPD and 0 = neither PD diagnosis. No participants in the sample were found to meet current criteria for a manic episode, and so this psychiatric comorbidity was dropped from subsequent analysis. No individuals with either PTSD or CPTSD were classed as harmful alcohol users (as opposed to alcohol-dependent), therefore logistic regression was not performed on this variable.

#### Sociodemographic and historical information

Demographic (age, ethnicity, immigration status, country of birth), and social (educational attainment, previous employment status, living situation) data were collected using a brief questionnaire developed for the purposes of the study. Clinical (previous mental health diagnoses) and forensic information (offence history) was also gathered as part of this questionnaire. Index offence and sentence length were collected from the Computer-National Offender Management Information System (C-NOMIS).

#### Statistical analysis

All statistical analyses were performed using STATA (version 15.1). Simple descriptive statistics (frequency distributions, chi-square analyses) were conducted to describe the sample and assess the prevalence of PTSD and CPTSD. Potentially salient covariates (age, ethnicity, educational qualification, relationship status, living status, employment status, immigration status, previous history of anxiety/depression), were identified from literature searches and explored using multinomial logistic regression. Those identified as independently associated with PTSD and/or CPTSD were then adjusted for in subsequent analyses. Two multinomial logistic regression models were then performed to calculate unadjusted and adjusted ORs regarding the likelihood of PTSD and CPTSD class membership, as compared to the sub-clinical group (the reference category): one to explore associations with trauma exposure characteristics, and the second to examine mental disorder comorbidity. Two additional multinomial logistic regression models were also conducted, in which the PTSD class was set as the reference group to determine whether (1) exposure to specific types of traumatic events or (2) specific mental disorders were associated with a diagnosis of CPTSD compared to a diagnosis of PTSD.

## Results

### Sample characteristics

Participants (*N* = 221) ranged in age from 18 to 54, with a median age of 30 (s.d. 9). Participant demographics are summarised in Table 1. The sample was ethnically diverse, and statistical analysis indicated no significant differences in age (*X*^2^ = 1.6, *p* = 0.45) or ethnicity (*X*^2^ = 5.3, *p* = 0.70) between our sample and the wider prison population, as reported in 2018 (HM Chief Inspector of Prisons, 2018). Our sample did differ from the wider prison site in that it included a smaller proportion of foreign nationals (14.5% *v.* 37.7%; *p* < 0.0001) (HM Chief Inspector of Prisons, 2018). The index offence(s) of our sample could not be compared due to a lack of reported data from the wider prison estate.

**Table 1.** Sample demographics

Characteristic *Total* (*N* *=* *221*)	Mean (s.d.) or *N* (Percentage)
Age	31.3 (9.0)
Ethnicity	
White British	87 (39.4)
White Other	34 (15.4)
Black African/Black Caribbean	56 (25.3)
South Asian	16 (7.2)
Mixed/Other	28 (12.7)
Nationality	
UK	188 (85.5)
Foreign National (including EU)	32 (14.5)
Employment status at the point of entry to prison	
Employed	105 (47.5)
Unemployed	116 (52.5)
Highest qualification^a^	
None	71 (32.4)
Any (GSCE/A-level; Degree; Certificate)	148 (67.6)
Age left school	15.4 (2.5)
Relationship status	
Single	184 (83.3)
Married/Cohabiting	37 (16.7)
Living situation (prior to prison)	
Fixed accommodation (incl. family)	177 (80.1)
No accommodation	44 (19.9)
Previous diagnosis of anxiety/depression	68 (30.8)
Index offence	
Violence against the person (e.g. ABH, GBH, Murder)	54 (24.5)
Weapons	16 (7.3)
Theft/Handling	39 (17.7)
Burglary	27 (12.3)
Robbery	10 (4.5)
Drugs	21 (9.5)
Fraud/Forgery	15 (6.8)
Sexual offences	10 (4.5)
Other (e.g. Motoring; Breach of court order)	21 (9.5)
Prev. hist. of violent offending	77 (34.8)
Sentence length >18 months^a^	70 (33.6)
Prisoner status^a^	
Newly sentenced	121 (55.3)
Transferred from other prison	56 (25.6)
Recalled to prison (license breach)	42 (19.2)

Demographic information for a sample of male sentenced prisoners (*N* = 221) residing in a large Category B prison in south London. Notes: Demographic information assessed using a self-report questionnaire. Forensic information (i.e. index offence, sentence length, prisoner status) gathered from prison records. History of violent offending measured using self-report questionnaire.

a Sample size <221 due to missing data.

### Prevalence of PTSD and CPTSD, sociodemographic and clinical covariates

The prevalence of current (past month) PTSD according to ICD-11 was 7.7% (*n* = 17, 95% CI 4.5–12). The prevalence of CPTSD was estimated at 16.7% (*n* = 37, 95% CI 12.1–22.3). The odds of PTSD class membership were not found to be increased by exposure to any of the covariates examined in the preliminary analysis, such as age, ethnicity, or academic qualifications (Table 2). The odds of CPTSD class membership were significantly higher amongst those who were not living in fixed accommodation prior to incarceration and those with a previous reported history of anxiety or depression. These variables were found to be independently associated with CPTSD class membership (aOR = 2.62, 95% CI = 1.2–5.8 and aOR = 2.28, 95% CI = 1.1–4.9, respectively). These covariates were therefore adjusted for in subsequent multivariate analyses.

**Table 2.** Sociodemographic, clinical covariates

Male Sentenced Prisoners (Total *N* = 221)	No PTSD/CPTSD *N* = 167	PTSD *N* = 17	CPTSD *N* = 37	PTSD		CPTSD		CPTSD		CPTSD	
Covariate	*N* (%)	*N* (%)	*N* (%)	OR^a^	CI	OR^a^	CI	AOR^b^	CI	AOR^c^	CI
Age	–	–	–	0.98	0.9–1.0	1.0	0.97–1.1	–	–	1.03	0.96–1.1
Ethnicity											
BME (*n* = 100)	76 (76)	10 (10)	14 (14)	1.7	0.6–4.7	0.7	0.4–1.5	–	–	0.4	0.1–1.4
White (*n* = 121)	91 (75.2)	7 (5.8)	23 (19)	–	–	–	–	–	–		
Employment status prior to prison											
Unemployed (*n* = 116)	81 (69.8)	11 (9.5)	24 (20.7)	1.9	0.7–5.5	2.0	0.9–4.1	–	–	1.0	0.3–3.4
Employed (*n* = 105)	86 (81.9)	6 (5.7)	13 (12.4)	–	–	–	–	–			
Educational qualifications											
None (*n* = 71)	48 (67.6)	6 (8.5)	17 (23.9)	1.3	0.5–3.8	2.1	0.99–4.3	–	–	1.6	0.5–5.1
Any (*n* = 148)	117 (79)	11 (7.4)	20 (13.5)	–	–	–	–	–	–		
Relationship status prior to prison											
Single (*n* = 184)	137 (74.5)	17 (9.2)	30 (16.3)	–	–	1.1	0.4–2.6	–	–	–	–
Married/cohabitating (*n* = 37)	30 (81)	0 (0)	7 (18.9)	–	–	–	–	–	–	–	–
Immigration status											
Foreign national (*n* = 32)	26 (81.2)	1 (3.1)	5 (15.6)	0.3	0.04–2.6	0.8	0.3–2.4	–	–	2.5	0.3–23.2
UK national (*n* = 188)	140 (74.5)	16 (8.5)	32 (17)	–	–	–	–	–	–		
Living status prior to prison											
Homeless (*n* = 44)	26 (59)	5 (11.4)	13 (29.6)	2.3	0.7–6.9	**2.9**	**1.3–6.5**	**2.8**	**1.3–6.3**	1.3	0.4**–**4.5
Stable accommodation (*n* = 177)	141 (79.7)	12 (6.8)	24 (13.6)	–	–	–	–	–	–		
Previous history of anxiety/depression (*n* = 68)	46 (67.7)	5 (7.4)	17 (25)	1.1	0.4**–**3.3	**2.2**	**1.1–4.6**	**2.2**	**1.0–4.5**	2.0	0.6–7.0
None reported	121 (79.1)	12 (7.8)	20 (13.1)	–	–	–	–	–	–		
Sentence length (>18 months)	52 (74.4)	9 (6.5)	25 (18.1)	1.6	0.5–4.4	0.9	0.4–1.9	–	–		
Sentence ⩽ 18 months	104 (75.4)	9 (6.5)	25 (18.1)	–	–	–	–	–	–		

Results of multinomial logistic regression examining historical, demographic, and clinical covariates. Notes: significant (*p* < 0.05) results are in bold.

a Reference group sub-clinical class.

b CPTSD results adjusted for significant covariates (living status, previous history of anxiety/depression).

c CPTSD results, PTSD class set as the reference category.

### Trauma characteristics and their association with PTSD/CPTSD

Individuals who had experienced their index trauma less than 1 year prior to assessment were over six times more likely to meet criteria for PTSD than individuals whose index events had occurred more distally (OR = 6.5, 95% CI = 1.9–22.2; see Table 3). The odds of PTSD class membership were also significantly increased by self-reported exposure to childhood physical abuse (OR = 3.6, 95% CI = 1.2–11.6) and emotional neglect (OR = 3, 95% CI = 1.1–8.3). Exposure to other childhood trauma types (e.g. verbal or sexual abuse), interpersonal and/or sexual violence, or an index event involving repeated exposure(s) was not significantly associated with PTSD class membership. Individuals who reported exposure to cumulative traumatisation in childhood or across the lifespan were also not significantly more likely to meet criteria for PTSD.

**Table 3.** Trauma antecedents

Total *N* = 221				PTSD		CPTSD		CPTSD		CPTSD	
Trauma Characteristic	No PTSD/CPTSD *N* = 167	PTSD *N* = 17	CPTSD *N* = 37	OR^a^	CI	OR^a^	CI	AOR^b^	CI	AOR^c^	CI
*Childhood trauma subtypes*	*N* (%)	*N* (%)	*N* (%)								
Childhood verbal abuse (*n* = 118)	79 (67)	10 (8.5)	29 (24.6)	1.6	0.6–4.4	**4.0**	**1.7–9.3**	**3.4**	**1.4–8.1**	2.5	0.7–9
Childhood physical abuse (*n* = 120)	79 (65.8)	13 (10.8)	28 (23.3)	**3.6**	**1.2–11.6**	**3.5**	**1.5–7.8**	**2.8**	**1.2–6.5**	0.8	0.2–3.4
Childhood sexual abuse (*n* = 32)	19 (59.4)	4 (12.5)	9 (28.1)	2.4	0.7–8.1	**2.5**	**1.1–6.1**	1.8	0.7–4.7	0.9	0.2–3.7
Childhood emotional neglect (*n* = 87)	54 (62.1)	10 (11.5)	23 (26.4)	**3.0**	**1.1–8.3**	**3.4**	**1.6–7.2**	**2.7**	**1.2–5.8**	0.9	0.3–3.3
Childhood physical neglect (*n* = 42)	19 (45.2)	3 (7.1)	20 (47.6)	1.7	0.4–6.3	**9.2**	**4.1–20.5**	**8.3**	**3.5–19.8**	**6**.**5**	**1.4–29.2**
*Trauma type* (*lifetime*)											
Interpersonal index trauma (*n* = 97)*	60 (61.9)	11 (11.3)	26 (26.8)	2.6	0.9–7.5	**3.4**	**1.6–7.4**	**3.1**	**1.4–6.8**	1.2	0.4–4.2
Sexual victimisation (*n* = 49)*	28 (57.1)	6 (12.2)	15 (30.6)	2.7	0.9–7.9	**3.4**	**1.6–7.3**	**2.6**	**1.1–5.9**	1.1	0.3–3.7
*Trauma timing*											
Index trauma occurring in childhood (*n* = 114)*	79 (69.3)	8 (7)	27 (23.7)	0.7	0.3–2.1	**2.3**	**1.0–5.1**	2.3	0.99–5.1	3.2	0.9–10.9
Index trauma occurring <1 year ago (*n* = 16)*	10 (62.5)	5 (31.2)	1 (6.3)	**6.5**	**1.9–22.2**	**0.4**	0.05–3.5	–	–	**0**.**07**	**0.007–0.6**
*Nature of exposure*											
Index trauma involving repeated exposure (*n* = 58)*	32 (55.2)	6 (10.3)	20 (34.5)	1.9	0.7–5.7	**4.2**	**2.0–8.9**	**4.0**	**1.8–8.7**	2.2	0.6–7.3
*Cumulative trauma*											
Childhood poly-victimisation (greater than 5 types) (*n* = 73)	44 (60.3)	6 (8.2)	23 (31.5)	1.5	0.5–4.4	**4.6**	**2.2–9.7**	**4.0**	**1.8–9.1**	3.5	0.9–13.3
Lifetime poly-victimisation (greater than 8 types) (*n* = 90)	58 (64.4)	9 (10)	23 (25.6)	2.1	0.8–5.8	**3.1**	**1.5–6.4**	**2.7**	**1.3–5.8**	1.4	0.4–4.5

Results of multinomial logistic regression examining associations between trauma antecedents and PTSD, CPTSD. Notes: *excluding *n* = 20 who did not identify an index trauma meeting criteria. Significant (*p* < 0.05) results are in bold.

a Reference group sub-clinical class.

b CPTSD results adjusted for significant covariates (living status, previous history of anxiety/depression).

c CPTSD results adjusted for significant covariates (living status, previous history of anxiety/depression), PTSD class set as the reference category.

In contrast, the unadjusted odds of CPTSD class membership were significantly increased by exposure to almost all of the trauma exposure characteristics explored (see Table 3). Individuals who reported exposure to verbal (aOR = 3.4, 95% CI = 1.4–8.1) or physical abuse (aOR = 2.8, 95% CI = 1.2–5.8) in childhood were significantly more likely to meet criteria for CPTSD, as were those who reported exposure to emotional (aOR = 2.7, 95% CI = 1.2–5.8) or physical neglect (aOR = 8.3, 95% CI = 3.5–19.8) following adjustment for covariates (living status and a previous history of anxiety/depression). No association between the experience of sexual abuse in childhood and CPTSD diagnosis was identified in adjusted analyses (aOR = 1.8, 95% CI = 0.7–4.7). Exposure to several other indicators of complex trauma across the lifespan was also strongly associated with CPTSD class membership. Individuals who identified an index trauma involving exposure to direct interpersonal violence (aOR = 3.1, 95% CI = 1.4–6.8), sexual victimisation (aOR = 2.6, 95% CI = 1.1–5.9), or which involved repeated or multiple exposure(s) (aOR = 4, 95% CI = 1.8–8.7) were all significantly more likely to meet criteria for CPTSD. Poly-victimisation was also significantly associated with CPTSD status. Specifically, the cumulative experience of more than five adverse experiences in childhood (aOR = 4, 95% CI = 1.8–9.1), or greater than eight trauma types across their lifetime (aOR = 2.7, 95% CI = 1.3–5.8) were both associated with CPTSD in adjusted analyses. The association between CPTSD class membership and exposure to an index event occurring in childhood (compared to one occurring after the age of 18) lost statistical significance once analysis was adjusted for covariates, although the association remained borderline significant (aOR = 2.3, 95% CI = 0.99–5.1).

Two characteristics of trauma exposure emerged as significant variables in differentiating CPTSD class membership from PTSD class membership. Those who reported exposure to childhood physical neglect were approximately six times more likely to belong to the CPTSD class than the PTSD class (aOR = 6.5, 95% CI = 1.4–29.2). Those who reported exposure to an index trauma occurring less than 1 year prior to assessment were significantly less likely to belong to the CPTSD class than the PTSD class (aOR = 0.07, 95% CI = 0.007–0.64).

### Psychiatric comorbidity

Individuals meeting criteria for probable generalised anxiety disorder were approximately six times more likely to belong to the PTSD class (compared to those without PTSD or CPTSD) (OR = 6.9, 95% CI = 2.4–20; see Table 4). Those with a cluster B personality disorder (OR = 4.9, 95% CI = 1.1–22.3) and alcohol dependence (OR = 3.2, 95% CI = 1.2–9.0) were also significantly more likely to meet criteria for PTSD. No significant associations were identified in unadjusted analyses between ICD-11 PTSD and depression, substance abuse, ADHD, or psychosis. Anxiety remained significantly associated with PTSD (aOR = 6.9, 95% CI = 2.0–23.5) following adjustment for other comorbid disorders.

**Table 4.** Psychiatric comorbidity

				PTSD		CPTSD		CPTSD		PTSD		CPTSD		CPTSD	
Disorder Total *N* = 221	No PTSD/CPTSD *N* = 167	PTSD *N* = 17	CPTSD *N* = 37	OR^a^	95% CI	OR^a^	95% CI	aOR^b^	95% CI	aOR^c^	95% CI	aOR^c^	95% CI	aOR^d^	95% CI
Anxiety (*n* = 72)	35 (48.6)	11 (15.3)	26 (36.1)	**6.9**	**2.4–20.0**	**8.9**	**4.0–19.8**	**7.5**	**3.3–17.1**	**6.9**	**2.0–23.5**	**3.4**	**1.2–9.4**	1.1	0.3–4.0
*Not present* (*n* *=* *149*)	132 (88.6)	6 (4)	11 (7.4)	–	–	–	–	–	–	–	–	–	–	–	–
Depression (*n* = 69)	38 (55)	6 (8.7)	25 (36.2)	1.8	0.6–5.3	**7.1**	**3.2–15.4**	**5.7**	**2.5–13.0**	0.7	0.2–2.6	**2.7**	**1.1–7.1**	3.6	0.98–13.0
*Not present* (*n* *=* *152*)	129 (84.9)	11 (7.2)	12 (7.9)	–	–	–	–	–	–	–	–	–	–	–	
Alcohol dependence (*n* = 58)	36 (62.1)	8 (13.8)	14 (24.1)	**3.2**	**1.2–9.0**	**2.2**	**1.0–4.7**	1.6	0.7–3.6	1.2	0.3–4.3	0.6	0.2–1.6	0.5	0.2–1.9
*Not present* (*n* *=* *163*)	131 (80.4)	9 (5.5)	23 (14.1)	–	–	–	–	–	–	–	–	–	–	–	–
Drug abuse (*n* = 75)	46 (61.3)	8 (10.7)	21 (28)	2.3	0.8–6.4	**3.4**	**1.7–7.2**	**2.6**	**1.1–6.1**	1.3	0.4–4.3	2.4	0.9–5.9	1.4	0.4–5.1
*Not present* (*n* *=* *146*)	121 (82.9)	9 (6.2)	16 (11)	–	–	–	–	–	–	–	–	–	–	–	–
Psychosis (*n* = 24)	12 (50)	1 (4.2)	11 (45.8)	0.8	0.1–6.5	**5.4**	**2.1–13.4**	**4.1**	**1.6–10.6**	0.2	0.02–2.3	1.5	0.5–4.6	6.1	0.7–53.1
*Not present* (*n* *=* *194*)	152 (78.4)	1 6(8.2)	26 (13.4)	–	–	–	–	–	–	–	–	–	–	–	–
ADHD (*n* = 76)	44 (57.9)	8 (10.5)	24 (31.6)	2.4	0.9–6.7	**5.1**	**2.4–10.8**	**4.3**	**1.9–9.3**	1.4	0.4–4.4	2.2	0.9–5.4	1.8	0.6–6.1
*Not present* (*n* *=* *143*)	121 (84.6)	9 (6.3)	13 (9.1)	–	–	–	–	–	–	–	–	–	–	–	–
Cluster B PD (*n* = 143)	99 (69.2)	15 (10.5)	29 (17)	**4.9**	**1.1–22.2**	**2.4**	**1.0–5.5**	2.0	0.8–4.8	3.6	0.7–17.2	1.5	0.6–4.0	0.4	0.1–2.4
*Not present* (*n* *=* *75*)	65 (86.7)	2 (2.7)	8 (10.7)	–	–	–	–	–	–	–	–	–	–	–	–

Results of multinomial logistic regression examining associations between CPTSD, PTSD and other mental disorders. Notes: All disorders assessed by validated tools via clinical interview. Significant (*p* < 0.05) results are in bold.

a Reference group sub-clinical class.

b Multivariate model adjusted for sociodemographic covariates (living status, previous history of anxiety/depression).

c Multivariate model adjusting for significant disorders in model and sociodemographic covariates, sub-clinical class as reference group.

d Multivariate model adjusting for significant disorders in model and sociodemographic covariates, PTSD class as reference group.

Substantially more psychiatric comorbidity was identified with CPTSD, with all measured disorders (anxiety, depression, substance misuse, alcohol dependence, psychosis, ADHD, cluster B personality disorders) significantly associated with CPTSD in unadjusted analyses (see Table 4). Following adjustment for historical covariates (living status prior to imprisonment, and a previous history of anxiety or depression), individuals meeting provisional criteria for depression (aOR = 5.7, 95% CI = 2.5–13), anxiety (aOR = 7.5, 95% CI = 3.3–17.1), psychosis (aOR = 4.1, 95% CI = 1.6–10.6), substance abuse (aOR = 2.6, 95% CI = 1.1–6.1), and ADHD (aOR = 4.3, 95% CI = 1.9–9.3), were all significantly more likely to belong to the CPTSD class (when compared to the sub-clinical class). Once other mental disorders were adjusted for, anxiety (aOR = 3.4, 95% CI = 1.2–9.4) and depression (aOR = 2.7, 95% CI = 1.1–7.1) remained significantly comorbid with CPTSD. The associations between CPTSD and substance misuse and ADHD were borderline statistically significant once other disorders were adjusted for.

Compared to individuals with PTSD, those with CPTSD were not more likely to be diagnosed with a comorbid mental disorder, except for depression, which was found to be over 3 times more likely to be associated with CPTSD than PTSD after adjustment for historical covariates, though the association was only borderline statistically significant (aOR = 3.6, 95% CI = 0.98–13.0).

## Discussion

This is the first study to report on the prevalence of ICD-11 PTSD and CPTSD in adult male prisoners using a disorder-specific measure. We had three objectives: to identify the prevalence of these disorders, to assess relationships with trauma characteristics, and to explore psychiatric comorbidity.

### Prevalence of PTSD and CPTSD

We found the rate of PTSD in our sample to be similar to previous meta-analytic estimates (Baranyi et al., 2018). The prevalence estimate of CPTSD, on the other hand, was substantially higher than that of PTSD, in line with previous studies from clinical populations and general population UK samples (Karatzias, Hyland, et al., 2019). The higher prevalence of CPTSD in a prison sample may be explained, in part, by high reported rates of complex and developmental trauma, both of which have been previously cited as risk factors for CPTSD development (Brewin et al., 2017; Karatzias et al., 2017).

### Relationship with traumatic experiences

We found that the recency of traumatic exposure increased the likelihood of belonging to the PTSD class, consistent with a recent study of a large, trauma-exposed adult population cohort in the UK (Karatzias, Hyland, et al., 2019). While low numbers in our PTSD group and wide confidence intervals limit conclusions, such a finding, paired with evidence from our sample that this characteristic was able to significantly distinguish those with CPTSD from those with PTSD, could imply that additional CPTSD symptoms develop as traumatic events and the responses thereto become more chronic.

Several characteristics of complex traumatisation increased the likelihood of belonging to the CPTSD class, but not the PTSD class when compared to those without symptoms. Exposure to traumatic events involving interpersonal and/or sexual violence, repeated trauma exposure, or cumulative exposure to multiple traumatic events over time, all increased the likelihood of belonging to the CPTSD class. Surprisingly, however, participants who experienced childhood sexual trauma were not more likely to meet criteria for CPTSD. Instead, our results indicated a particularly strong relationship between childhood physical neglect and CPTSD, and it was the only trauma characteristic which was more likely to be reported by individuals with CPTSD compared to those with PTSD, in line with a recent study (Frost, Hyland, Shevlin, & Murphy, 2020). Such a finding could reflect a retrospective reporting bias with men being less likely to report victimisation, particularly sexual victimisation, and more comfortable to disclose experiences of neglect (Priebe & Svedin, 2008). In sum, our findings provide additional support to the idea that complex trauma exposure (i.e. trauma which is prolonged and/or repeated, or consists of multiple forms), will confer greater risk for CPTSD (Brewin et al., 2017).

### Psychiatric comorbidity

We found that psychiatric comorbidity with ICD-11 PTSD was reduced, compared to both ICD-10 and DSM-5 estimates, in line with previous research (Brewin et al., 2017). This suggests that the WHO's efforts to produce a more specific set of diagnostic symptoms to describe trauma-related distress have, at least for PTSD, been partially effective. Previously reported high rates of psychiatric comorbidity with DSM PTSD may therefore be due, in part, to a broad DSM-5 diagnostic definition which shares similar clinical features with other disorders such as sleep disturbances, irritability, and loss of interest in activities previously enjoyed (Barbano et al., 2019; Facer-Irwin et al., 2019). None of these items is present in the ICD-11 PTSD classification (Brewin et al., 2017). Nonetheless, three disorders were associated with PTSD in unadjusted analyses. Those with PTSD were more likely to also screen positive for anxiety, alcohol dependence, and a cluster B personality disorder compared to those without PTSD or CPTSD. PTSD is just one disorder which can arise as a consequence of trauma (Green et al., 2010), is more likely to arise in individuals with pre-existing disorders (Brewin, Andrews, & Valentine, 2000) and increases the likelihood of development of other disorders (Breslau, 2009).

A diagnosis of CPTSD was found to be comorbid with multiple disorders in unadjusted analyses and remained significantly associated with several mental disorders (e.g. depression, substance misuse, psychosis, ADHD) following adjustment for potential confounders. Complex developmental trauma is associated with an increased risk of multiple deleterious psychiatric outcomes in adulthood, including CPTSD (Cloitre et al., 2019; Karatzias, Hyland, et al., 2019). The DSO features of the CPSD construct may overlap with other clinical presentations, such as depressive and cluster B personality disorder features. We were not able to examine specific associations between BPD and CPTSD in our study but found no significant association with cluster B personality disorders in adjusted analyses. Several studies using latent class and network analyses have supported the construct validity of PTSD and CPTSD and identified key distinctions from depression, anxiety, and cluster B personality disorders (Frost et al., 2020; Knefel et al., 2019). However, other studies have highlighted that discrete boundaries may be less clear, particularly within multiply-traumatised or complex clinical samples (Gilbar, 2020; Jowett, Karatzias, Shevlin, & Albert, 2020).

### Strengths and limitations

Our study is the first to examine the prevalence and correlates of ICD-11 PTSD and CPTSD diagnoses among sentenced adult male prisoners. Previous research examining CPTSD in prison settings has often modelled symptoms from scales not specifically designed to assess this disorder (Facer-Irwin et al., 2019), and the use of a standardised, validated instrument to measure these newly defined constructs was a clear strength of this study. The study also benefitted from a moderately-sized, ethnically diverse sample which was found to be representative of the wider male prison estate.

Nonetheless, the ITQ remains a relatively new scale not previously used within prisons. Future validation work within this population is needed. Furthermore, the use of a self-report method of symptom endorsement to establish ‘probable’ PTSD/CPTSD diagnoses may have increased the risk of symptom overreporting and inflated diagnostic rates. While a measure indicating atypical responses is not currently included in the ITQ, precluding its direct assessment in this study, this may represent an important consideration for clinicians and researchers in forensic settings. The development of a clinician-administered diagnostic interview for ICD-11 PTSD and CPTSD is ongoing (Brewin et al., 2017), and is a necessary next-step for research examining such constructs, particularly within settings where the risk of over-inflation of CPTSD estimates is likely.

The decision to use symptom screening tools with cut-offs in lieu of diagnostic interviews to assess for other mental disorders (i.e. depression, anxiety, substance and alcohol misuse, ADHD) was taken primarily because of the pressures of time inherent in prison research and to reduce the potential burden on respondents; however, such a decision also likely inflated estimates of these disorders (Fazel et al., 2016). Similarly, we relied on retrospective measures of traumatic experiences, and recent research has highlighted that risks associated with exposure to ACE vary considerably depending on whether retrospective or prospective accounts are used (Baldwin, Reuben, Newbury, & Danese, 2019).

Finally, our moderately-sized sample, and the small number of those with ‘simple’ PTSD, likely limited the power of the analyses to investigate relationships with this construct or draw comparisons with CPTSD, as indicated by some of the wide confidence intervals noted in the analysis. As our investigation was an exploratory study with multiple comparisons, future dedicated studies with pre-planned outcomes are needed to confirm our results.

### Implications and conclusions

Within our UK sample of male sentenced prisoners, PTSD and CPTSD remained distinguishable disorders with distinct clinical correlates, a finding with important implications for the assessment and treatment of trauma-related psychopathology in prison settings. Improving the identification of trauma symptomology and provision of evidence-based treatments are emphasised as the foundations of effective trauma-informed service delivery, the implementation of which is increasingly being recognised as crucial within prison settings (Branson, Baetz, Horwitz, & Hoagwood, 2017). The lower prevalence of PTSD in our study compared to previous DSM estimates may indicate improved diagnostic precision when assessing PTSD according to ICD-11 criteria, which is valuable within a population where the prevalence of all mental disorders is high (Fazel et al., 2016). Post-hoc analyses of participants' healthcare records indicated that the overwhelming majority of those who met criteria for PTSD (82.4%, *n* = 14) or CPTSD (75.7%, *n* = 28) were not detected by prison healthcare staff. While the rates of identification of most mental disorders in prisons are low, one previous study similarly found prisoners with PTSD to have particularly significant unmet treatment needs (Jakobowitz et al., 2017). This highlights that PTSD and CPTSD are often under-identified in prison settings, perhaps due to the presence of other, co-occurring disorders or the minimal recognition of prisoners' trauma histories by healthcare professionals. The lack of formal recognition of CPTSD as a diagnosis until very recently may have led to misdiagnosis and have had significant ramifications for prisoners' access to services and appropriate psychological treatment.

Our findings highlight that the identification and treatment of PTSD and CPTSD in prison settings should be made a clinical and research priority, given that they appear to represent distinct groups with different clinical treatment needs or associated risks. Emerging evidence from community samples suggests that many trauma-focused interventions may not be as effective at treating CPTSD symptoms (Karatzias, Murphy, et al., 2019; Mahoney, Karatzias, & Hutton, 2019). Trauma-focused psychological interventions delivered in prison settings have reportedly limited efficacy (Yoon, Slade, & Fazel, 2017), and there is currently no literature on specific interventions for prisoners with CPTSD. Preliminary evidence from UK clinical and community samples has also highlighted that individuals with CPTSD may be at an increased risk of high-risk outcomes including suicidality, self-harm, and aggression (Hyland, Shevlin, Fyvie, & Karatzias, 2018; Karatzias, Hyland, et al., 2019). Yet, CPTSD remains empirically understudied regarding its association with such adverse behavioural outcomes in prison settings (Facer-Irwin et al., 2019). Findings from the present study highlight the increasing need for research which differentiates between these two disorders, to examine their potentially distinct roles in adverse outcomes within UK prisons.

## Supplementary material

For supplementary material accompanying this paper visit https://doi.org/10.1017/S0033291720004936. click here to view supplementary material
