# Supplementary material for: PTSD and complex PTSD in sentenced male prisoners in the UK: prevalence, trauma antecedents, and psychiatric comorbidities
Source: Psychol Med. 2021 Jan 12;52(13):2794–804. doi: 10.1017/S0033291720004936 (PMC9647511; doi:10.1017/S0033291720004936)
Supplement: Supplementary file 1 [file S0033291720004936sup.zip › S0033291720004936sup001.docx]

Supplementary material Figure 2: Antecedent trauma in PTSD vs CPTSD

The index traumas categorized as interpersonal assault were defined as those involving actions of another person intentionally causing or threatening death, injury or sexual violence. This could be either directly experienced by participant (i.e. direct interpersonal) or witnessed happening to someone else (i.e. witnessed interpersonal). A direct accident was defined as an index trauma categorized as an accident or an illness directly experienced by the participant, and witnessing accident was defined as an accident or illness that the participant witnessed occurring to other(s). Other trauma categories were network trauma (i.e. a traumatic event which affected someone in the participant’s network that they learned details of, but had not directly experienced or witnessed) or other trauma (i.e. a trauma that did not fit into any other category) – in this sample, these were either perpetrator traumas or exposure to repeated details of trauma through work. Index trauma categorisations and definitions taken from Lewis and colleagues (2019).
