# Supplementary material for: PTSD and complex PTSD in sentenced male prisoners in the UK: prevalence, trauma antecedents, and psychiatric comorbidities
Source: Psychol Med. 2021 Jan 12;52(13):2794–804. doi: 10.1017/S0033291720004936 (PMC9647511; doi:10.1017/S0033291720004936)
Supplement: Supplementary file 1 [file S0033291720004936sup.zip › S0033291720004936sup002.docx]

**80 excluded following approach**

Insufficient English (n=39)

Lacked capacity/too unwell (n=11)

Risk concerns (n=4)

Did not meet criteria (n=9)

Could not take part (awaiting release or deportation) (n=17)

**237 took part in a clinical interview**

**N= 221 Final sample included in analysis**

5 withdrew consent during data collection

11 excluded due to too much missing data

9075 Prisoners admitted into custody during sampling period

**432 approached to participate**

**264 consented to take part**

**3477 Eligible to take part (Sentenced, age 18-55)**

**88 declined**

**27 did not complete an interview**

Transferred (n=6)

Released (n=7)

Withdrew consent (n=9)

Could not be located after repeated attempts (n=4)

Increased risk (n=1)

Excluded by researcher due to risk (n=1)

446 identified to approach

14 transferred/released prior to approach

Supplementary material Figure 1: Flowchart of recruitment
