# Supplementary material for: PTSD and complex PTSD in sentenced male prisoners in the UK: prevalence, trauma antecedents, and psychiatric comorbidities
Source: Psychol Med. 2021 Jan 12;52(13):2794–804. doi: 10.1017/S0033291720004936 (PMC9647511; doi:10.1017/S0033291720004936)
Supplement: Supplementary file 1 [file S0033291720004936sup.zip › S0033291720004936sup003.docx]

Supplementary material 2: Variables assessed in paper

| Variable | Definition | Source | Observations |
| --- | --- | --- | --- |
| ***Trauma characteristics*** |  |  |  |
| Trauma timing (age) | Index trauma occurring in childhood (before 18) or adulthood | ITQ | 200 ^a^ |
| Trauma recency | Index trauma occurring within 1 year of interview | ITQ | 200 ^a^ |
| Interpersonal trauma | Index trauma involving direct experience of interpersonal violence | ITQ | 200 ^a^ |
| Sexual victimization | Any previous history of sexual trauma | ACE, LEC | 221 |
| Prolonged, repeated exposure | Index trauma involving repeated, prolonged exposure | ITQ | 200 ^a^ |
| Cumulative trauma (childhood) | Exposure to 5 or more types of childhood trauma/adversity | ACE | 221 |
| Cumulative trauma  (lifetime) | Exposure to more than 8 types of trauma across the lifetime (i.e. one-above the mean number of types experienced by sample) | LEC | 221 |
| ***Comorbidities*** |  |  |  |
| Depression | Current (2 weeks) severe depressive symptoms (scores ≥15) | PHQ-9 | 221 |
| Anxiety | Current (2 weeks) severe anxiety symptoms (scores ≥15) | GAD-7 | 221 |
| Harmful alcohol use | Current (past year) harmful alcohol abuse (scores ≥16) | AUDIT | 221 |
| Alcohol dependence | Current (past year) alcohol dependence (scores ≥20) | AUDIT | 221 |
| Substance abuse (substantial) | Current (12 months) substantial substance abuse problems (scores≥6) | DAST-10 | 221 |
| Psychotic disorder | Current (past month) psychotic disorder | MINI | 218 ^b^ |
| ADHD | “Probable” ADHD diagnosis (past 6 months) | ASRS screen | 219 ^b^ |
| Cluster B PD | Met criteria for ASPD and/or BPD | MINI/SCID-5 | 218 ^b^ |

a excluding n=21 participants who did not identify a qualifying index trauma b missing data due to incomplete interviews
